# Methods for designing patient-specific templates for optimized fitting of autologous bone augmentations in alveolar cleft osteoplasty

**DOI:** 10.1186/s13005-026-00613-1

**Published:** 2026-03-24

**Authors:** Tom A. Schröder, Philipp Sembdner, Erik Selbmann, Uwe Teicher, Korn Paula, Michaela Buckova, Farahzadi Samaneh, Pradel Winnie, Günter Lauer, Anas Ben Achour

**Affiliations:** 1 Department of Oral and Maxillofacial Surgery, Faculty of Medicine and University Hospital Carl Gustav Carus, TUD Dresden University of Technology, Fetscherstraße 74, 01307 Dresden, Germany; 2 Department of Mechanical Engineering, Institute of Machine Elements and Machine Design, Technische Universität Dresden, 01062 Dresden, Germany; 3 Fraunhofer Institute for Machine Tools and Forming Technology IWU, Nöthnitzer Straße 44, 01187 Dresden, Germany; 4 WHZ Westsächsische Hochschule Zwickau, Kornmarkt 1, 08056 Zwickau, Germany

**Keywords:** Alveolar cleft osteoplasty, Surgery, Bone augmentation, Cutting guides, Digitalization in medicine

## Abstract

**Background:**

Following the current gold standard, autologous cancellous bone is used as part of alveolar cleft osteoplasty. To fill the bone defects, autologous bone material is harvested from the iliac crest using manual tools such as shepard chisels or trephine drills. The bone augmentations obtained in this way have a simple geometry, usually cylinders, and must then be manually adapted to the defect to be filled by the surgeon using surgical forceps and scissors. There are no established routines for the manufacturing of patient-specific, cost-effective surgical cutting guides. However, the accuracy of fit of the augmentations plays an important role in the healing process.

**Methods:**

This paper focuses on a concept for the creation of necessary sequenced incision geometries based on 3D X-ray data of alveolar cleft defects.

**Results:**

As a result, a procedure is to be developed for the preoperative design of individualised surgical cutting guides based on image data. We described a workflow to segment the cleft defect using reverse engineering from Cone-beam computed tomography (CBCT) data. The data was further processed and a keyhole contour was created. A stamping guide and a cutting guide were then derived. The stamping guides were scaled 5, 10 and 15% larger than the defect. In addition, two half-shells were produced, which will be used to investigate the clamping forces and the biological consequences in a follow-up study.

**Conclusions:**

This article presents a developed routine for creating patient-specific templates and demonstrates its feasibility.

## Background

With an incidence of 1 in 700 births, cleft lip and palate are the most frequent facial malformations [1]. Surgical closure of the alveolar cleft is performed usually between the ages of 7 and 11 [2]. The primary aim of alveolar cleft osteoplasty is to restore the dental arch in order to achieve complete dental rehabilitation [1, 2]. According to the current gold standard, the bone defect is filled manually by using spongiosa cylinders made of autologous iliac crest [3]. First, the autologous bone grafts are harvested from the iliac crest using one of two standard manual tools - Shepard’s chisel or trephine drill. Secondly, the harvested bone grafts must be shaped with hand tools (forceps, scissors, scalpels) before they are inserted into the cleft defect. The workflow presented in this paper can be used to collect more information on success factors and potentially prevent additional operations. Technical developments in digitalisation and the production of medical devices are increasingly enabling the customisation of implants, tools and surgical accessories. A systematic investigation of effect sizes and threshold values in the transplantation of iliac crest cancellous bone in the case of cleft osteoplasty depends on preoperative 3D imaging and can only be successful with its methodical interpretation. In recent years, methods and tools for calculating virtual anatomical 3D models from imaging data have become established, whereby a discrete 3D surface model is generated by segmenting the area of interest in the image data and subsequently creating isosurfaces [4]. Nevertheless, segmentation in image data is still a time-consuming process, which varies depending on the body region and the associated complexity of the bony structure as well as the quality of the image data, which is reduced by artefacts, respectively. Patient-specific surgical tools are frequently used in cutting and drilling guides, for example for joint implantation or the implantation of dental implants. Various biocompatible materials made of metal, plastic or ceramic are available.

For complex, delicate bony areas such as the jaw region, especially for alveolar cleft the state of the art in science and technology does not yet offer an adequate solution to interactively support or even automate segmentation for the creation of customised templates.

## Methods

### 3D data processing

The alveolar cleft defect of the exemplary patient for the development of the routines was first recorded using a CBCT (3D Acciutomo MCT-1, J. Morita MFG. Corp., Kyoto, Japan). Scans were acquired with an isotropic voxel size of 0.160 mm (0.160 × 0.160 × 0.160 mm). Slice thickness and slice interval were both set to 0.160 mm. The in-plane pixel spacing was 0.160 × 0.160 mm. The acquisition matrix was 503 × 529 pixels per slice, with a total of 529 slices per volume. The reconstructed volume radius was 40 mm (corresponding to an approximate field of view of 80 mm in diameter). Imaging parameters were set to 86 kV and 6 mA, with an exposure time of 17.5 s, resulting in a tube load of 105 mAs. The CTDIvol (CTDIw) was 9.5 mGy. The source-to-patient distance was 500 mm and the source-to-detector distance was 710 mm. Image reconstruction was performed using the manufacturer’s default reconstruction filter (FBP; reconstruction filter G_001). The patients CBCT data was used with approval from the institutional ethics committee (Reference No. BO-EK-111022021).

**Fig. 1:**
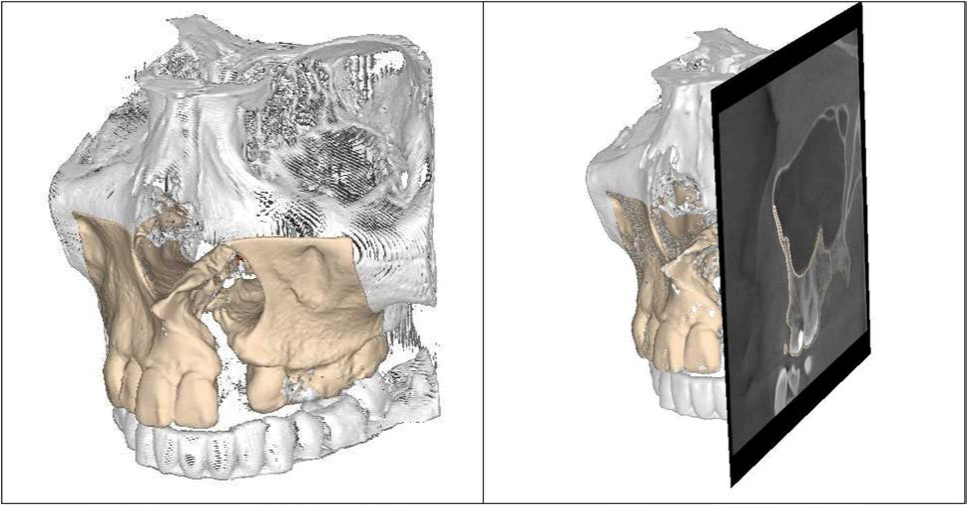
superimposition of the generated surface model onto the original image data within CTinA: coronal on the left, coronal with sagittal cutting plane on the right

The defect area was then segmented in a semi-automatic workflow using reverse engineering methods and the CTinA software solution from the Chair of Virtual Product Development at TU Dresden [5–7]. The resulting 3D model was then processed using Geomagic Wrap software, imported as 3D model into commercially available design software via reverse engineering and further processed (SolidWorks 2020, Dassault Systèmes, Frankreich). A constant gray-value threshold corresponding to approximately 1000 (12-bit dataset) was applied to differentiate mineralized bone from surrounding soft tissue. Surface reconstruction was performed using the Marching Cubes algorithm to generate a triangulated isosurface model. The threshold selection was carried out manually by the primary operator to achieve optimal representation of cortical bone structures. One trained operator conducted the segmentation, while a second experienced operator from the medical field performed an independent manual review for quality control. Inter- and intra-operator variability were minimized by using a standardized threshold and a defined review process. However, formal statistical variability analysis was not performed. The segmentation process required approximately 30 min, including data import and threshold definition (semi-automatic workflow), followed by approximately two hours of manual post-processing to refine contours and prepare the model for further CAD processing using Geomagic Wrap. The performed quality control included visual slice-by-slice inspection of the CBCT data with superimposition of the generated surface model onto the original image data within CTinA (Fig. 1).

**Fig. 2:**
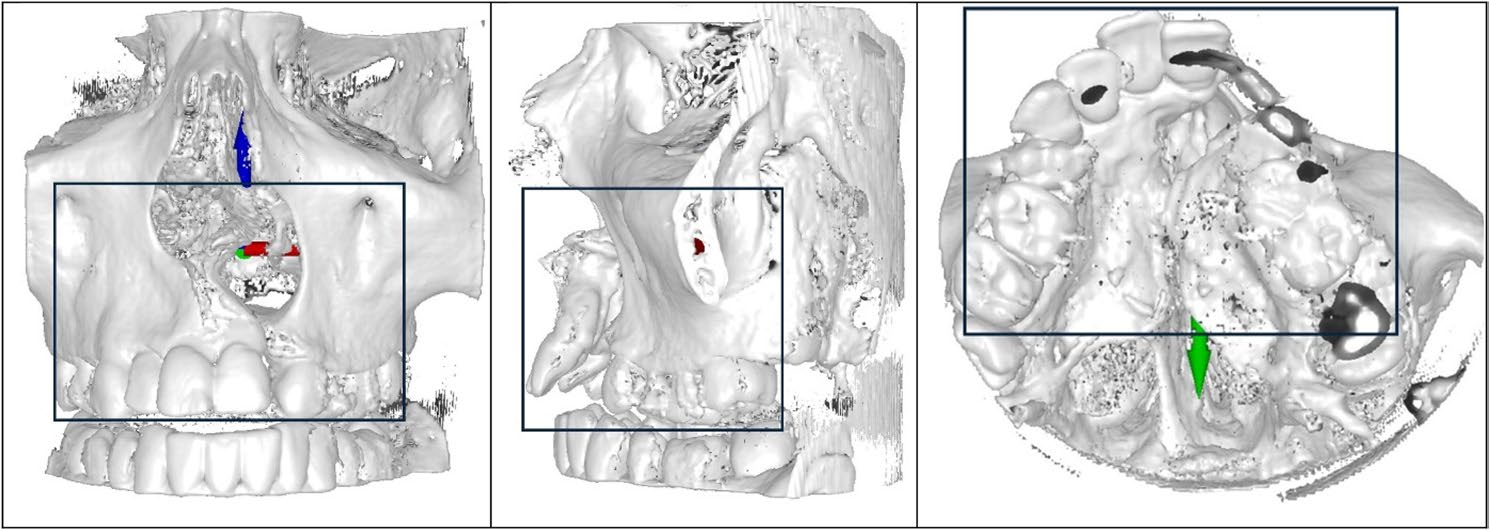
Definition of the region of interest (ROI): coronal on the left, sagittal in the middle, and axial on the right

The region of interest (ROI) was defined on the initially segmented, unprocessed dataset by trained medical personnel (second operator) based on clinical relevance of the defect morphology. The defined ROI was documented using screenshots (Fig. 2) and subsequently transferred manually to the 3D model by defining cutting planes and contour boundaries, ensuring anatomically and surgically consistent defect representation.

A reference plane was manually defined on the imported 3D models based on the classic surgical approaches to the defect. The establishment of this reference plane should be conducted as a central step in collaboration with surgical support. An adjustment to the individual defect morphology of the patient is recommended, so that no universal plane definition is deemed meaningful. The virtual model was rotated according to the surgeon’s viewpoint, and a plane was defined by the intersection of three points at the outer anatomical bone shell of the defect which are chosen manually. The plane thus created is almost perpendicular to the surgeon’s virtual line of sight. A segmentation of the defect starting from the surgical access and extending into depth is possible via the automatic plan-parallel displacement with defined distances. A contour representing the access to the undercut area was defined in this cutting plane where the smallest contour is visible using the semi-automatic spline function of the software. This so-called keyhole contour was then exported from the 3D model of the skull and used to design tools and devices. In a first step, the contour was modified in order to generate a cutting template that can be produced by milling and is based on the principle of a stamping press. The contour processing for subtractive manufacturing required smoothing especially inner radii < 0.1 mm and contours with high gradients of change (such as sharp edges) so that production with precision tools would be feasible from an economical standpoint. However, the minimal changes to the contour were classified as acceptable deviations of the real contour. This cutting template is subject to a number of requirements:

- The inner edges of the cutting tool reproduce the contour of the keyhole true to scale.
- The wall of the template must have high rigidity and wear resistance, especially at the cutting edge.
- Use of a biocompatible material with sterilizability.
- Can be manufactured using a cutting process (milling).
- Good handling of the template.

The cutting templates were first machined by milling from solid material 1.4404 / 316 L and subsequently ground using a sinker EDM process. The fabrication was performed according to the 3D data set, and tolerances below 0.1 mm were maintained. After production, the components were cleaned in an ultrasonic bath, disinfected, and sterilized using a laboratory autoclave.

It is also necessary to generate the final bone shape for the anterior closure of the defect on the 3D model of the skull. Cylindrical cutting tubes with the same diameter as the bone grafts were designed for this purpose. When closing the defect, the surgical procedure was taken into account during positioning and virtually formed using Boolean operation. This virtual geometry is then used to generate cutting guides to optimise the contact surfaces to the defect-bordering maxillary bone. For the practical use of cutting guides, material-related hygiene and mechanical requirements are just as essential as a flawless cut and easy loading and unloading of the guides. For the design of the guide, technical concepts were discussed with experienced oral and maxillofacial surgeons and a design was created based on their experience.

A) Development of a test bench for measuring the clamping force.

**Fig. 3:**
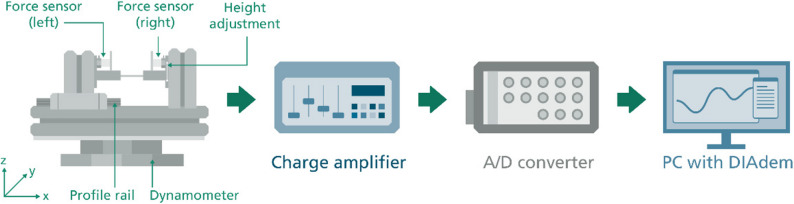
Test setup for measuring the clamping force, A/D = analogue-to-digital

In order to reduce the need for animal testing, it is necessary to analyse the question of optimum clamping force by running in-vitro tests. Part of this second study, which is still to be carried out, consists of clamping the patient-specific bone grafts in physiological representations of the defect and keeping them alive with physiological nutrient solution. The contact surfaces of the grafts are then examined for damage caused by clamping force. In preparation for this in-vitro study, a routine for the production of tools and equipment was first developed and implemented. In a first step, the dimensions of the contour were scaled up around its centre of the contour area in the centre plane. In accordance with the assessment of achievable accuracy in manual trimming with, for example, pliers or similar tools a 5, 10, 15% oversizing was selected as scaling factors and stamping guides equivalent to those described above were produced. Oversizing of the bone cylinders is necessary to achieve clamping effect. Different degrees of oversizing were tested to identify the optimal scaling that produces an effective clamping mechanism while preventing bone resorption due to excessive mechanical stress exceeding critical thresholds. To simplify the handling of these stamping templates in the laboratory, an additional stamping counterpart was designed and manufactured using a separation process (milling) and removal (electrical discharge machining). This stamping counterpart is intended to support the contouring process of the bone cylinders with a sharp-edged inner contour and a positioning aid in the form of semi-cylindrical support surfaces. The final element required for the in-vitro investigation of the clamping force is a three-dimensional image of the defect. The basic test setup (Fig. 3) and the critical value for the clamping force to be analysed without damaging the bone specify the following requirements:

- Use of biocompatible materials.
- Design must enable sensor-based detection of clamping forces à Installation in measuring device or integration of sensors must be possible.
- Easy cleaning and sterilisation of the contact surfaces.
- Can be manufactured using standard production processes.
- Medium to high surface quality.
- Implanted grafts must be surrounded by culture medium.

To meet these requirements, so-called graft matrices were designed based on the original keyhole contour as test carriers in the form of half-shells that can be joined together. These half shells are necessary so that the corresponding clamping forces can be measured when inserting the bone cylinders with the aid of a test set-up shown in Fig. 3. For this purpose, the half-shells are placed in the test set-up and pressed together with a high preload force. The connected highly dynamic and high-resolution force sensor is used to measure the effective splitting force, which represents the counterforce to the clamping force. Once this splitting force has been recorded, the half-shells must be joined together while maintaining the clamping forces and without leakage. The volume of the reshaped defect must also be filled with culture medium. For this purpose, the graft matrix is provided with four screw points and a circumferential nut for installing a rubber seal.

## Results

### Cutting guides

**Fig. 4:**
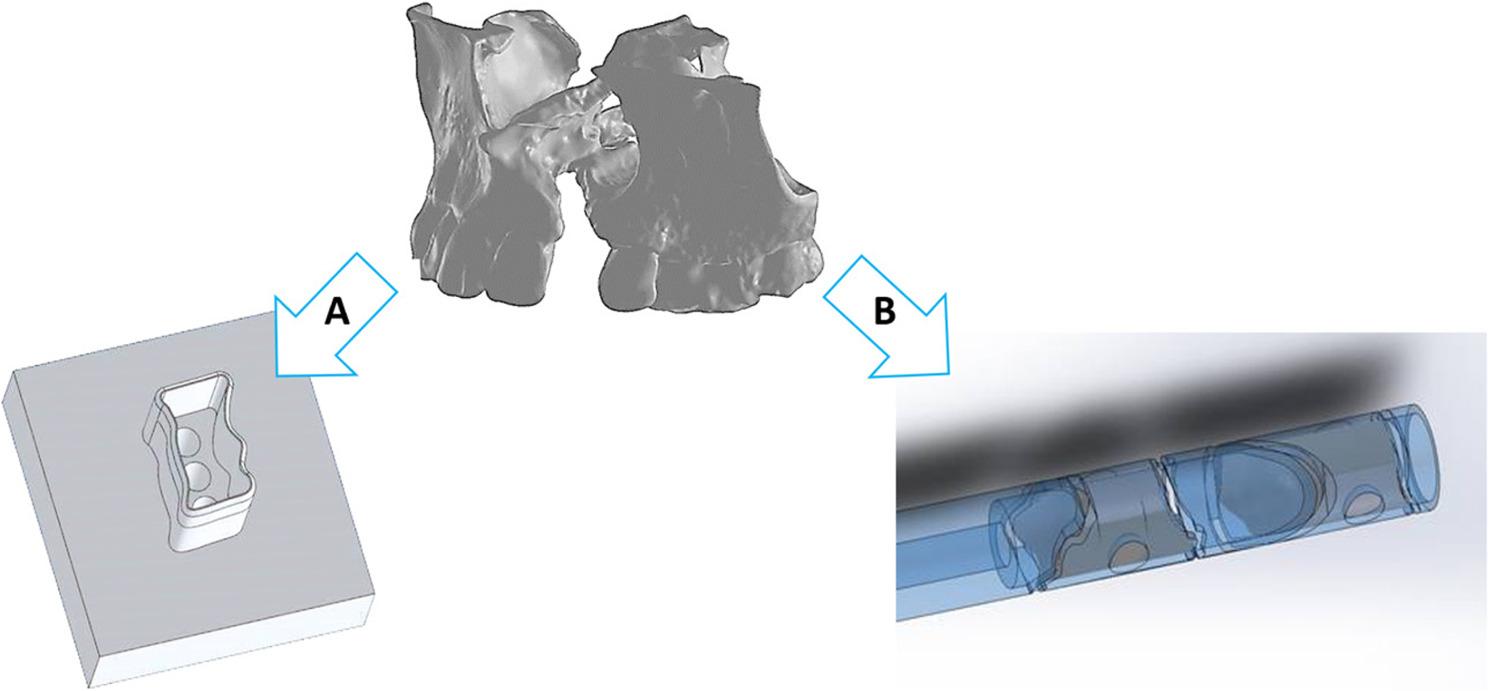
(**A**) Stamping guide; (**B**) 3D retraction of the preoperative bone defect and a derivation of a cutting guide for defect closure.

The first prototype of the stamping template (Fig. 4A) was tested in a laboratory study using pig cadaver bone to evaluate the influence of the harvesting parameters when obtaining bone material from the iliac crest. As can be seen in Fig. 4A, a high degree of shape retention of the augmentation can be achieved using the stamping tool despite the relatively high porosity of the bone cylinder. However, when using the prototype stamping tool, it became clear that a rear hole should be provided to make it easier to remove the stamped bone grafts from the template (ejector). These holes were added to the design before the oversized stamping templates were produced. Figure 4B shows the 3D retraction of the preoperative bone defect and a derivation of a cutting tube. The designed cutting tube enables the cutting of two cylinders in one tube and thus contributes to resource efficiency. The cutting lines are twisted around the axis of rotation of the tube in order to ensure the stability of the tube even with a thin-walled design. In addition, large holes are drilled on both sides at the height of the axis of rotation to hold the bone cylinders in the starting position during cutting. For loading, the grafts can be inserted into the front part of the cutting tube. The number of required cylinders will depend on the length of the grafts as well as the cleft volume itself. A hole is provided in the handle of the cutting tube for unloading the cutted pieces. An ejector can be inserted through this hole, which pushes the bone pieces out of the tube. Figure 4 shows two methods of preoperative computer-assisted planning with the derivation of a stamping template 4 A and a cutting tube 4B respectively. Both methods can be used in addition to each other or alone. An advantage of the combination is usually in the presence of undercuts, as these can be taken into account to a limited extent via the stamping template if fitting is still necessary.

## Stamping templates and in-vitro clamping templates

**Fig. 5:**
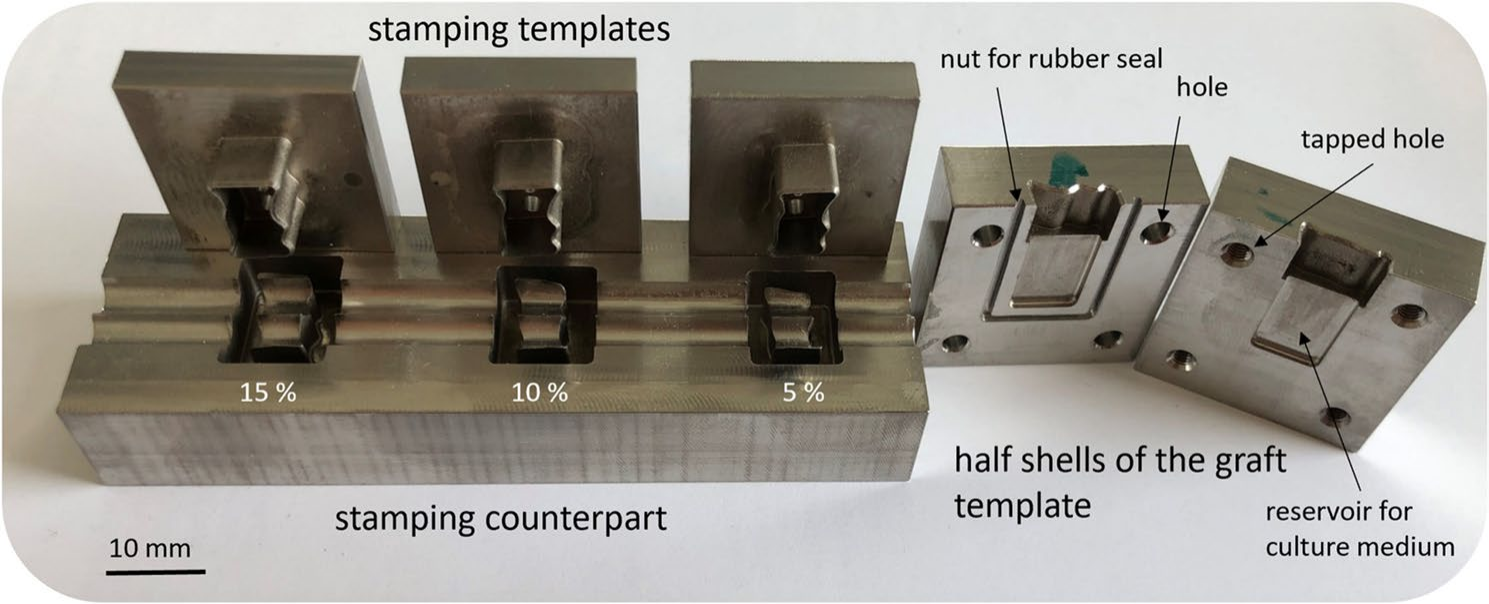
Left: Matrix with stamps in different size scaling, Right: Half shells of the graft template to investigate clamping forces

**Fig. 6:**
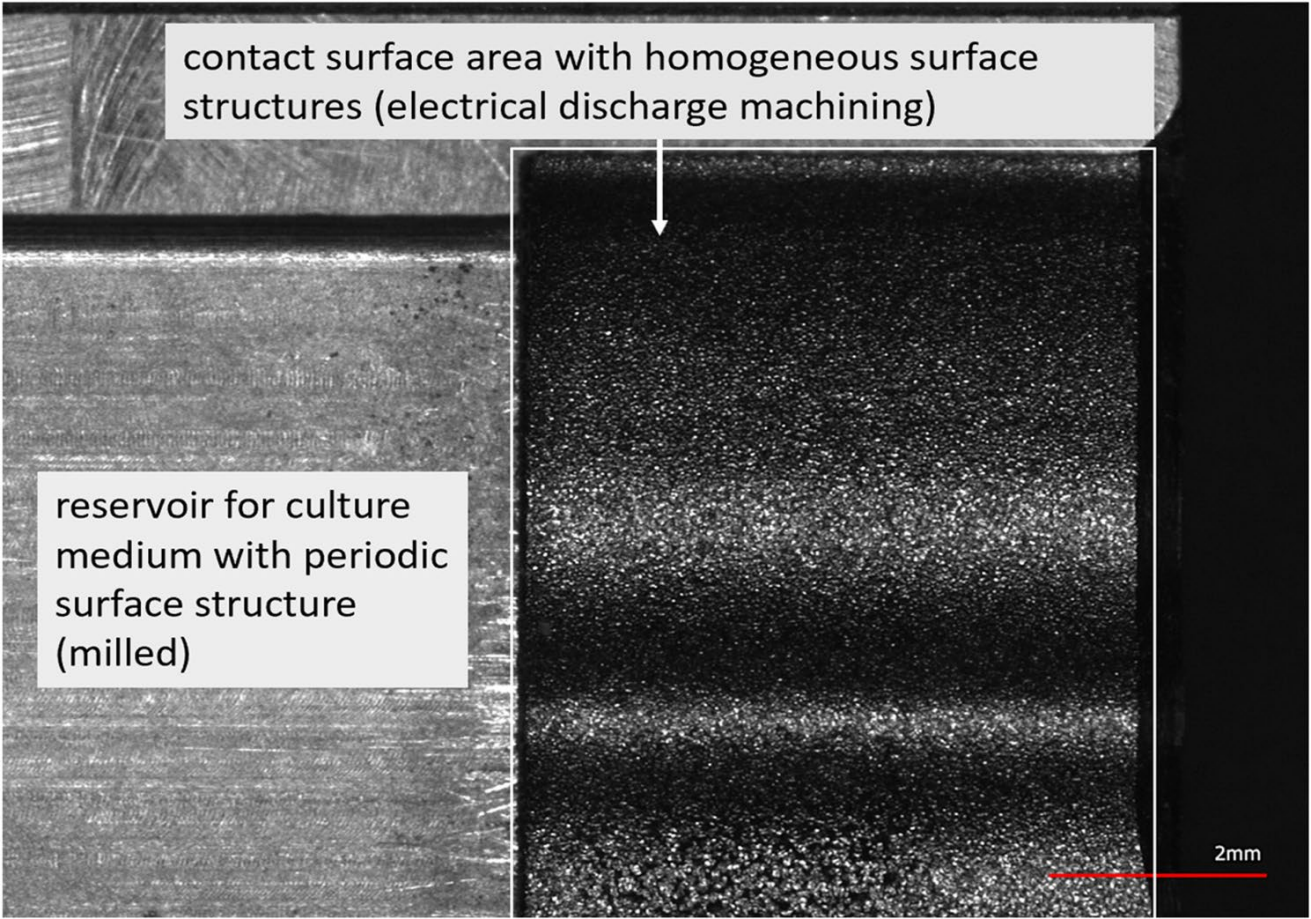
Microscope image (top view) of the graft template with visible differences in the surface structure

Figure 5 shows the three defined oversized stamping templates, the fabricated counterpart and the half shells of the graft template. The material of choice was EN ISO 1.4404 / AISI 316 L (stainless steel), a material that has proven itself in medical applications. The stamping templates can be positioned on the upper edge and cut the bone smoothly as a result of a precision fit between the inner contour of the stamp and the outer contour of the defect in the counterpart. However, positioning proved to be sensitive to errors when loaded with bone cylinders. To avoid incorrect positioning during stamping, an additional guiding system for the stamping template should therefore be added. The transplant cutting guides produced also fulfil the aforementioned requirements. Figure 5 also shows two half-shells that were constructed to investigate the clamping forces and their effects on cell vitality. In addition (Fig. 6), a matt and predominantly homogeneous surface structure was found in the area of the contact surfaces due to the manufacturing process. This was created by the electrical discharge machining of the contour. Compared to a surface that is only milled this surface structure is more representative than a technical simulation of the surface of the cleft borders and should therefore be favoured. In summary, the tools presented still need to be analyzed in the planned in-vitro study described above.

## Discussion

According to the current gold standard, the bone defect is filled with cancellous bone cylinders from autologous iliac crest, as previously mentioned [3]. The harvested bone grafts must then be shaped by the surgeon using hand tools (forceps, scissors, scalpels) before being inserted into the cleft. A relevant disadvantage is the low grade of standardization and the high dependency on the experience of the surgeon required preparing the cancellous bone cylinder [8]. A systematic review including 25 studies and a total of 422 patients showed that the described current gold standard achieves a long-term filling rate of the alveolar cleft defect of approximately 62% (95% CI 54.3 to 69.6) [9]. In many patients, augmentation treatments become necessary once again in the course of dental rehabilitation. The above-mentioned therapeutic gold standard for alveolar clefts describes a manual preparation of the cancellous bone cylinders prior to bone grafting [3]. This depends to a large extent on the expertise of the surgeon when it comes to fitting the bone cylinders into the cleft defect. The quantity of bone cylinders is also limited due to the morbidity of the harvest. Another aspect is the locally occurring pressure peaks within the transplanted cancellous bone. These can occur if the graft is too tightly clamped in the defect. Bone it-self is a pressure-sensitive tissue that reacts to excessive pressure with resorption [10]. Oversizing the bone cylinders (necessary for clamping) can therefore lead to resorption of the surrounding bone if critical limits are exceeded. The limits have not yet been definitively proven but they will certainly have an impact a complete bony reconstruction of the defect. We already described a holistic approach for the identification of success factors in secondary cleft osteoplasty in our previous work [11]. As there is no animal model for hereditary alveolar clefts and ethical considerations are increasingly focussed on reducing and avoiding animal testing, such studies require the development of alternative in vitro methods that allow equally reliable transferability of results. The templates are to be used during surgery for correct and efficient individualisation of the cancellous bone cylinders. The use of cutting guides will enable consistently good surgical results, regardless of the surgeon’s level of experience. The workflow is described using the example of an alveolar cleft. In addition, exemplary templates of realistic cleft defects are created for in-vitro studies. Sensors are also integrated into these models. The sensors will be used to systematically determine limit values for acceptable oversizing of the augmentations. The pressure load on the implants can also be recorded and used to describe limit values for avoiding pressure resorptions. 3D cleft measurement is currently not part of the pre-operative routine, but is carried out for complicated cleft defects and to determine the tooth structure in a large number of patients. However, if better long-term results in terms of the cleft volume are achieved with preparation using cutting templates, the inclusion of preoperative CBCT diagnostics in the clinical standard is conceivable. Furthermore, in-tensive efforts are being made to enable the three-dimensional measurement of cleft defects using X-ray-free imaging. One approach that we are working on is to measure defects using ultrasound and corresponding data processing. This method would make it possible to translate the defect geometry and manufacture the described templates and counterparts. The production of the stamping or cutting guides does not present any economic or time constraints, as the complexity of the guides can be standardised down to the patient-specific part. The model generation for the patient-specific part is largely CAD-supported and can also be automated if the procedure is implemented economically. The use of AI methods in image processing has increased in the last years, particularly on the research side, in order to increase the quality of the segmentation on the one hand and to endeavour to automate the segmentation on the other [12]. These methods, such as the Active Shape Model (ASM), can be used to produce customised templates for joint implantation in the knee area, for example [13]. For this purpose, parameterised model descriptions and thus individualised adaptations, e.g. via an ASM/SSM model, are suitable for future further developments.

## Conclusions

The article presented the developed routine, proved its feasibility and demonstrated the benefits using a specific application example. There is potential for improvement in the following aspects:

- The workflow for creating the model were carried out manually, but offer potential for automated solutions within the process steps using image processing methods in combination with machine learning and artificial intelligence approaches.
- Algorithms for surface processing can be developed for semi-automated template generation in combination with a parametric system to be integrated in order to reduce the processing time and thus ultimately also address the aspect of the economic efficiency of the process.
- The process can also be used to provide recommendations for the surgical procedure, e.g. virtually via software solutions in the sense of a knowledge-based assistance system. In the future, this could lead to a knowledge base to achieve an equally good surgical result regardless of the surgeon’s experience.
- Transferability to other areas of the body.
- A significant advantage that can be expected is the surgical procedure. It is no longer necessary to prepare the graft only manually. The operation is therefore standardised, which also reduces operating time.
- The removal morbidity of the iliac crest cancellous bone can also be reduced, as the required amount of augmentation material can be determined in advance.

In a more long-term perspective (5–10 years), a consistent further development of the routines described in this work would also be conceivable as an operating theatre-related production of patient-specific tools such as the described punching templates or other surgical accessories. However, scientific and legal issues relating to data acquisition and processing, production processes in synchronisation with operating procedures, liability and hygienic conditions must be clarified and corresponding capacities built up at care centres. Advanced and flexible individualisation in this form can be a crucial factor in improving medical and diversified care. However, in view of the current global crisis, it must be ensured that the customised components produced can be recycled. Otherwise, realisation will fail.

## Data Availability

not applicable.
